# Myocardial oxygenation is maintained during hypoxia when combined with apnea – a cardiovascular MR study

**DOI:** 10.1002/phy2.98

**Published:** 2013-10-11

**Authors:** Dominik P Guensch, Kady Fischer, Jacqueline A Flewitt, Matthias G Friedrich

**Affiliations:** 1 Departments of Cardiac Sciences and Radiology, Stephenson Cardiovascular MR Centre at the Libin Cardiovascular Institute of Alberta, University of CalgaryCalgary, Alberta, Canada; 2 Philippa & Marvin Carsley CMR-Centre at the Montreal Heart Institute, Université de MontréalMontreal, Quebec, Canada

**Keywords:** BOLD, carbon dioxide, CMR, hypoxia, myocardial oxygenation

## Abstract

Oxygenation-sensitive (OS) cardiovascular magnetic resonance (CMR) is used to noninvasively measure myocardial oxygenation changes during pharmacologic vasodilation. The use of breathing maneuvers with OS CMR for diagnostic purposes has been recently proposed based on the vasodilatory effect of Co_2_, which can be enhanced by the additive effect of mild hypoxia. This study seeks to investigate this synergistic concept on coronary arteriolar resistance with OS CMR. In nine anesthetized swine, normoxemic and mild hypoxemic arterial partial pressure of oxygen (Pao_2_) levels (100 and 80 mmHg) were targeted with three arterial partial pressure of carbon dioxide (Paco_2_) levels of 30, 40, and 50 mmHg. During a 60-sec apnea from the set baselines, OS T2*-weighted gradient echo steady-state free precession (SSFP) cine series were obtained in a clinical 1.5T magnetic resonance imaging (MRI) system. Arterial blood gases were acquired prior to and after apnea. Changes in global myocardial signal intensity (SI) were measured. Although a greater drop in arterial oxygen saturation (SaO_2_) was observed in the hypoxemic baselines, myocardial SI increased or was maintained during apnea in all levels (*n* = 6). An observed decrease in left ventricular blood pool SI was correlated with the drop in SaO_2_. Corrected for the arterial desaturation, the calculated SI increase attributable to the increase in myocardial blood flow was greater in the hypoxemic levels. Both the changes in Paco_2_ and Pao_2_ were correlated with myocardial SI changes at normoxemia, yet not at hypoxemic levels. Using OS CMR, we found evidence that myocardial oxygenation is preserved during hypoxia when combined with Co_2_-increasing maneuvers, indicating synergistic effects of hypoxemia and hypercapnia on myocardial blood flow.

## Introduction

Noninvasive imaging techniques are increasingly used for the diagnosis of cardiovascular diseases (Schwitter and Arai 2011; Cannon et al. 2013). As prevalent disorders such as coronary artery disease present as a mismatch of oxygen supply and demand in the heart, imaging techniques that assess myocardial oxygenation may be of crucial importance for future diagnostic approaches (Friedrich and Karamitsos 2013). Oxygenation-sensitive (OS) cardiovascular magnetic resonance (CMR) imaging allows for measuring changes in myocardial oxygenation during pharmacological vasodilation. The sensitivity to changes of oxygenation exploits the inherent signal reducing properties of deoxygenated hemoglobin (Hb) resulting in local inhomogeneities in the magnetic field (Ogawa et al. 1990) and the resulting loss in signal intensity (SI) in CMR images acquired with OS protocols. Vice versa, an increase in the measured signal represents a decrease in dHb and implies an increase in regional oxygenation.

Myocardial oxygenation and thus the SI in images using this blood oxygen level dependent (BOLD) effect are determined by the coronary blood supply controlling the volume of Hb, the oxygen saturation of the blood, and the oxygen extraction by the tissue. In situations with decreasing saturation, maintenance or increases in OS-SI are compensated by coronary flow. In order to change the supply, current protocols include the administration of a pharmacological vasodilator, commonly adenosine or dipyridamole (Niemi et al. 1996; Egred et al. 2007).

However, local metabolites also have a strong effect on the intrinsic control of coronary blood flow, in particular o_2_ and co_2_ (Feigl 1983; Deussen et al. 2012). While metabolites control local myocardial blood flow in close relationship to the metabolic requirements, systemic changes of o_2_ and co_2_ will result in an uncoupling of myocardial blood flow from its metabolic demand, similar to the effects of pharmacologic vasodilators. Several studies have confirmed that high co_2_ levels induce higher coronary flow rates, while low levels reduce blood flow (Markwalder and Starling 1913; Broten et al. 1991).

All vasodilating agents have a pronounced effect on the microcirculation by reducing the resistance to blood flow. Specifically, arterioles of under 150 microns are the most sensitive to metabolites, adenosine, and dipyridamole (Chilian et al. 1989; Chilian and Layne 1990; Kuo and Chancellor 1995). The dilation of arterioles is significant for tissue oxygenation through the subsequent increase of capillary recruitment and capillary perfusion pressure (Honig et al. 1980; Rhoades and Bell 2009). Because of this function, these agents are highly suitable for OS imaging as the majority of the BOLD effect originates from the high proportion of deoxygenated blood in the capillaries (Kassab et al. 1994; Wacker et al. 1999, 2003).

This study investigates the use of metabolite controlled blood supply. Previously, we demonstrated in both healthy animals and volunteers that undergoing a breath-hold results in maintenance or an increase of the myocardial oxygenation, even in the presence of blood deoxygenation (Guensch et al. 2013a). An increase in myocardial oxygenation despite desaturation of the arterial blood can only be explained by a compensatory increase in myocardial blood flow. However, these studies were conducted using normal baseline blood gas levels. The current study uses the breath-hold technique from a wider range of oxygen and carbon dioxide tension baselines that may be seen in some patient cohorts, testing the robustness of the technique and expanding on the idea of the BOLD dependence on blood gases by tying in the augmenting effect of hypoxemia on hypercapnic vasodilation (Broten et al. 1991).

The aim of this study was to exploit the vasoactive response to carbon dioxide and oxygen tensions to measure the ability of the heart to maintain myocardial oxygenation using OS CMR.

## Materials and Methods

### Anesthesia and animal protocol

Nine juvenile male pigs (24.3 ± 0.2 kg) were premedicated with ketamine (600 mg), midazolam (10 mg), and fentanyl (2 mg) i.m., and then anesthetized with intravenous thiopental (20–25 mg/kg). Animals were intubated with an endotracheal tube (ID 5.5–6 mm) and ventilated with a Harvard Ventilator. For the MR exam, extended tubing (12 m) was used for the ventilation and intravenous drugs to connect to the equipment outside of the MR suite. Anesthesia was maintained with an intravenous drip (1–3 mg/h midazolam, 1.6–4.8 mg/h fentanyl) and nitrous oxide/isoflurane (0.6–1.5%). Lidocaine (60 mg/h) was continuously infused to prevent arrhythmia. The left jugular and femoral vein were cannulated for intravenous infusions, and the right carotid artery and the femoral artery were cannulated for access to measure invasive blood pressure and arterial blood gases. Anesthesia and hemodynamics were monitored by inspiratory and end-tidal gas measurements, three-channel ECG, invasive blood pressure, and arterial blood gases.

### Experimental protocol

**Figure 1:**
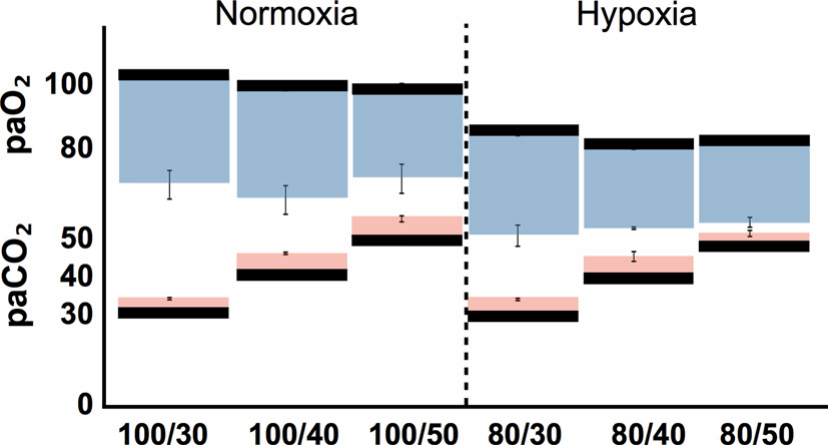
Baseline blood gas values (bold lines) and changes of Pao_2_ and Paco_2_ (*n* = 6). After a 60-sec breath-hold, Pao_2_ (blue) decreased significantly for all levels while the significant change in Paco_2_ (red) was positive (*P* < 0.05).

Six baseline blood gas levels were targeted (Arterial partial pressure of oxygen [Pao_2_]: 100 and 80 mmHg; Arterial partial pressure of carbon dioxide [Paco_2_]: 30, 40, and 50 mmHg) by modulating the ratio of inspiratory o_2_/N_2_O gas fraction and the ventilation rate (Fig. 1). This way hypocapnia and hypercapnia were assessed in combination with normoxemia or hypoxemia in addition to a normal blood gas level previously published by our group (Guensch et al. 2013b). Once a level was attained, a 60-sec breath-hold was induced by pausing ventilation at end expiration. Immediately after resuming ventilation, a second arterial blood sample was taken to determine the changes in blood gas levels over the breath-hold. Blood gases were utilized to calculate the approximate arterial Hb saturation using a dissociation curve tool (Varjavand 2000) based on the equations of Kelman (1966) and Severinghaus (1979).

### Imaging protocol

All imaging was performed in a clinical 1.5T magnetic resonance imaging (MRI) system using a body matrix coil (Avanto®, Siemens Healthcare, Erlangen, Germany). Single-slice BOLD-sensitive steady-state free precession (SSFP) cine images were acquired continuously throughout a 60-sec breath-hold of one mid left ventricular short axis (slice thickness 10 mm, echo time 2.78 msec, repetition time 44.48 msec, flip angle 90°, field of view 280 × 157.5, matrix 128 × 72). Each cine series was composed of 20 phases covering the entire cardiac cycle, obtained by retrospective ECG gating.

### Image analysis

Mean SI of the left ventricular (LV) myocardium and arterial blood was obtained from images using certified software for CMR image analysis (cmr^42^, Circle Cardiovascular Imaging Inc., Calgary, AB, Canada). Myocardial SI was defined by the manual tracing of endocardial and epicardial contours, and the arterial blood by a region of interest in the LV lumen. For each cine, the area under the curve (AUC) was calculated from the SI of all 20 phases to provide a single value for each acquisition. The SI from the first two cines of the breath-hold were compared to the final two cines and expressed as percent change SI measured across a 60-sec breath-hold. The percent change in SI is reported for both the myocardium (MyoSI) and the left ventricular blood pool (LVbpSI). To compensate for the two competing effects of arterial desaturation and increase in CO_2_-mediated blood flow, the difference between LV-blood pool SI change and myocardial SI change is reported as calculated SI change (SI_calc_). This myocardial SI corrected for desaturation calculates as SI_calc_ = MyoSI (%) – LVbpSI (%).

### Statistical analysis

Changes in values at baseline and after a breath-hold were compared using paired t-tests. Changes in SI of both the myocardium and blood pool were compared to the changes in Paco_2_, Pao_2_, heart rate (HR), MAP (mean arterial blood pressure) and calculated arterial oxygen saturation (SaO_2_) with linear correlation and multiple regression analysis. Statistical analysis was considered significant if *P < 0.05.

### Ethics statement

This study was conducted in accordance with the “*Guide to the Care and Use of Experimental Animals”* by the Canadian Council on Animal Care. It was approved by the local “*Animal Care and Use Board*” and the institutional ethics committee.

## Results

For each level, there were six successful subjects due to two premature deaths and one to a preexisting cardiac abnormality. All images were of sufficient image quality and none were excluded. Table1 shows the observed changes of cardiovascular parameters induced by apnea under different blood gas levels.

**Table 1:** Mean ± SEM of the changes in cardiovascular parameters during a 60-sec breath-hold starting from different arterial blood gas levels (*n* = 6, *P < 0.05)

Start level		ΔHR	ΔMAP

Pao_2_	Paco_2_		
100	30	7.9 ± 5.9	−15.6 ± 2.2*
	40	5.2 ± 4.7	−9.8 ± 3.4*
	50	9.4 ± 4.1	−9.3 ± 1.7*
80	30	15.1 ± 9.6	−18.0 ± 2.0*
	40	5.8 ± 3.1	−17.5 ± 3.7*
	50	8.8 ± 2.8*	−14.5 ± 4.0*

A significant drop in MAP was observed in all levels, while a change in HR was only seen during the breath-hold in the hypoxemic and hypercapnic level. HR, heart rate (beats/min); MAP, mean arterial blood pressure (mmHg).

### Image analysis

**Figure 2:**
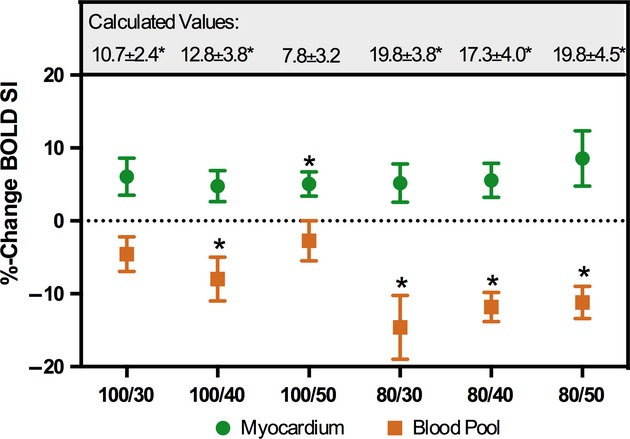
Percent change signal intensity (SI) of the myocardium (green circles) during apnea. Myocardial SI (oxygenation) was maintained or increased from baseline in all levels. At hypoxemic states, the percent change SI of the blood pool (orange squares) was greater and shows a significant decrease. The corrected hyperemia-induced signal was therefore greater in the hypoxemic states. (*n* = 6, *P < 0.05, from baseline image).

The initial baseline SI did not differ between the different levels and the percent change SI is the reported value. The MyoSI showed a similar trend of increasing SI during breath-holds of at least 5.2% for each level (Fig. 2). On the other hand the LVbpSI decreased, but the degree of change differed between some levels. The decrease was not as pronounced in the normoxemic states (Pao_2_ 100 mmHg), yet a larger and significant drop of at least 11% (*P < 0.02) was observed in the hypoxemic states (Pao_2_ 80 mmHg). The same effect was seen in the SaO_2_ calculation. There was an increase in the calculated myocardial SI of at least 17% in the hypoxemic states (*P < 0.01) and at least 10% for the levels 100/30 and 100/40 (*P < 0.05), with 100/50 to be the only level with a nonsignificant increase (+7.8%, *P* = 0.08).

### Blood gas analysis

Paco_2_ and Pao_2_ levels were analyzed for all blood samples (*n* = 6) except for the post–breath-hold oxygen tension from one animal of the 80/40 level resulting in *n* = 5. Analysis of the arterial blood samples (Fig. 1) from each level showed that Paco_2_ increased significantly while the oxygen partial pressures all decreased by a minimum of 25 mmHg after a 60-sec breath-hold. These changes were consistent among all the levels. Like the changes in LVbpSI, there was a greater decrease in calculated SaO_2_ in the hypoxemic levels compared to the normoxemic levels.

### Relationship of signal intensity to blood gas levels

**Figure 3:**
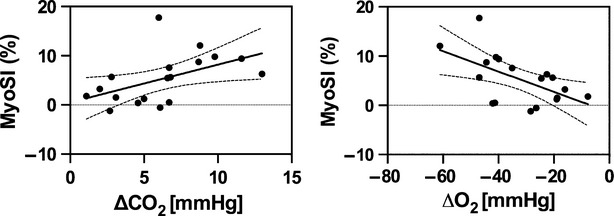
Relationship between signal intensity changes with arterial blood gases at normoxemic baseline states: A positive correlation was observed between the myocardial SI/oxygenation changes and the change in Paco_2_ (*r* = 0.50, *P* = 0.03, *n* = 18; left panel), while a negative correlation was encountered with the change in Pao_2_ (*r* = 0.57, *P* = 0.01, *n* = 18; right panel).

**Figure 4:**
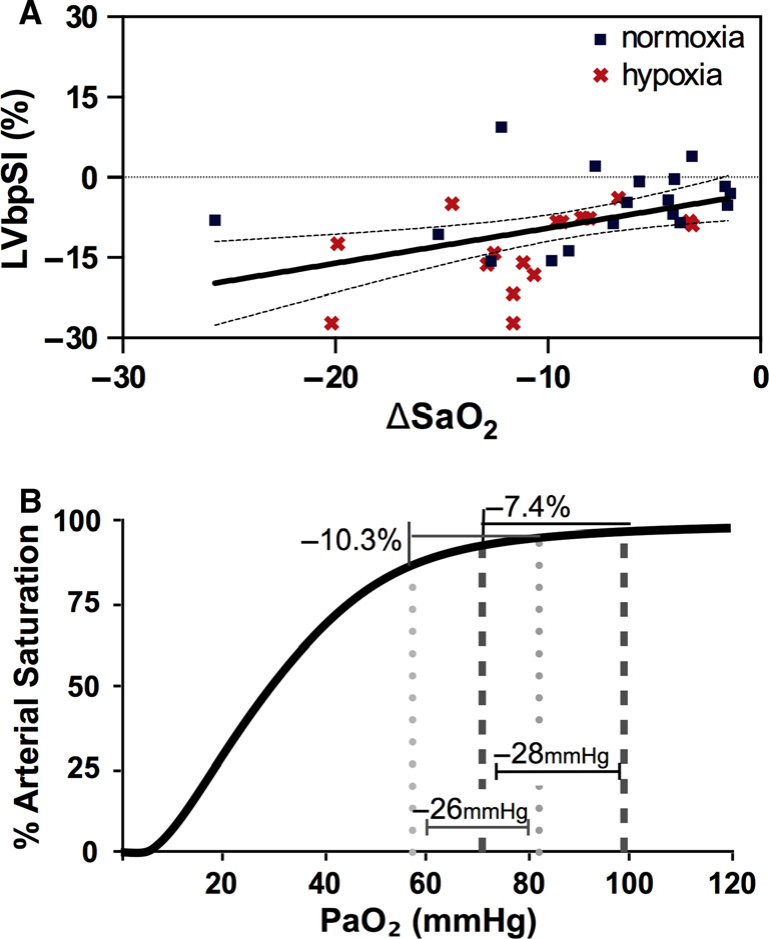
Relationship of Hb Saturation and Blood Pool SI: A moderate correlation was observed between the percent change in SI of the left ventricular arterial blood (LVbpSI) and the calculated changes in SaO_2_ (*r* = 0.46, *P* < 0.01, *n* = 35; upper panel). The measured baseline and post–breath-hold oxygen tension for both the normoxemic (100/50, dark gray) and the hypoxemic (80/50, light gray) levels are simulated on a Hb saturation curve shifted for the hypercapnic states (lower panel) showing the hypoxemic baseline's position on the steeper slope of the curve with its greater decrease in SaO_2_ after a similar decrease in Pao_2_ in comparison to normoxemia.

Blood pool SI was found to be moderately correlated to the change in SaO_2_ (*r* = 0.46, *P* < 0.01, Fig. 4). Analysis of the myocardial SI was divided into hypoxemic and normoxemic levels. In the hypoxemic levels no relationship was observed between myocardial SI and any blood gases. For the normoxemic levels, correlations (Fig. 3) were present between myocardial SI and both Paco_2_ (*r* = 0.50, *P* = 0.03) and Pao_2_ (*r* = −0.57, *P* = 0.01). Multiple regression showed that both of these parameters could be combined to explain the changes in SI (R^2^ = 0.42, *F*_2,15_ = 5.43, *P* = 0.02).

### Relationship of signal intensity to cardiovascular parameters

There was no correlation between myocardial BOLD-SI changes and HR or mean arterial blood pressure. However, there was a moderate negative correlation between HR and left ventricular blood pool SI (*r* = 0.47, *P* = 0.049).

## Discussion

Our OS CMR data reflect an additive effect of hypoxemia and co_2_ increase on coronary vasodilation during a breath-hold when corrected for desaturation in the arterial blood of the LV cavity. Myocardial oxygenation is preserved even during the more pronounced hypoxemic states. Our endpoints, however, refer to repeated measurements and thus are not sensitive to stable systematic confounders. Most importantly, all experiments have been performed under steady conditions with respect to oxygen consumption. The observed changes therefore reflect the tissue response to the transient effects of the breath-hold.

Oxygenation is vital for viable tissue, even more so in cardiac muscle due to the high oxygen demands of the beating heart. The role of the coronary response to changes of myocardial oxygen demand has been extensively studied on systemic, coronary, and microvascular levels. Myocardial oxygenation changes are reflected as a result of both, the regional oxygen extraction fraction and the myocardial perfusion reserve (Ogawa et al. 1990; Wacker et al. 1999; Zhang et al. 2007).

Metabolite- and adenosine-mediated vasodilation occurs in the arterioles instead of the capillaries making them primarily responsible for the flow conductance as they create the majority of the coronary vascular resistance (Chilian and Layne 1990; Kuo and Chancellor 1995; Muller et al. 1996; Komaru et al. 2000). Yet, one of the final factors in increasing tissue perfusion is the regulation of capillary function in modulating the blood flow to the tissue (Duling and Klitzman 1980). Normally only about 55–64% of the microvasculature is perfused allowing for a large functional reserve when required (Weiss and Conway 1985). This ability to increase the signal is representative of the vasodilating reserve available from resting conditions.

### Normoxemia

Unlike other OS CMR studies, we utilized the intrinsic vasoactive properties of co_2_ and o_2_. Consistent with previous observations in animals and humans showing a correlation between the changes in blood co_2_ and SI in OS images during breathing maneuvers with normal blood gas levels at baseline (Guensch et al. 2013b), we found the same trend when a 60-sec breath-hold was induced from a range of Paco_2_ levels at normoxemia, even in a hypercapnic environment. In addition to the co_2_ relationship, the multiple regression analysis showed that the oxygen levels in conjunction with co_2_ can explain the changes in myocardial SI for the normoxemic states. This is expected as the arterial tension of both o_2_ and co_2_ have been reported to modulate coronary flow (Case and Greenberg 1976; Broten et al. 1991).

### Hypoxemia

Broten et al. (1991) demonstrated that vasodilatory effects are potentiated by the combination of hypoxia and hypercapnia, as the response of coronary flow to changes in oxygen tensions is greater at high co_2_ coronary tension than at low co_2_, and vice versa for oxygen. These findings are integral to our study, and describe the mechanisms responsible for the observed changes in OS-SI. Starting at normoxemia (Pao_2_ 100 mmHg), Figure 1 shows that by the end of the breath-hold Pao_2_ decreased ∼30 mmHg to mild hypoxemic states. However, this study went even further by assessing three levels which underwent a breath-hold from an already stable but mild hypoxemia. Thus after the breath-hold, Pao_2_ levels were measured to be markedly hypoxic in the range of 50–60 mmHg, but myocardial oxygenation was still maintained similar to the normoxemic states. This is of importance for considerations regarding a clinical application, in which a variety of different abnormal baseline blood gas levels may be encountered. As shown in Figure 4A, the correlation of the blood pool SI with the calculated saturation supports the idea that the signal of the lumen reflects the actual Hb saturation levels.

### Corrected hyperemia induced signal

Our observations are consistent with the assumption that the discrepancy between changes of blood pool and myocardial oxygenation is explained by a significantly increased blood supply as we account for arterial Hb desaturation during the breath-hold. SI of the blood pool in the ventricular lumen is independent from perfusion or metabolic effects and is principally a representation of the dHb in the blood. Consequently, analysis of the SI from both the myocardium and the blood pool of the same MR image reflect the compensatory mechanisms of the coronary and microvascular system during systemic Hb-oxygenation changes. As the blood in the left ventricle is the sole blood source for the coronary arteries, the same desaturation as in the LV has to be assumed in the coronary system, which can be accounted for if the extent of desaturation is known. Thus we subtracted the relative drop in signal in the left ventricular blood pool from the change in myocardial signal to isolate the perfusion changes from the SaO_2_ changes. Interestingly, the values corrected for arterial desaturation are larger for the hypoxemic states. During hypoxemic states, the myocardium was able to maintain SI and thus oxygenation, even with a much greater decrease in arterial o_2_ saturation (Fig. 2). Therefore, one can infer that although performing an effective breath-hold from a mild hypoxemic baseline, myocardial oxygenation is still preserved and the increase in myocardial blood flow is greater for the hypoxic states in order to counteract the effects of desaturation. This is supported by findings of other groups which demonstrated that hypoxia has a synergistic effect to hypercapnia in animal and human studies (Feigl 1983; Broten et al. 1991).

A decrease in myocardial SI would, however, be expected with severe desaturation. In our model, we see a negative correlation between the change in myocardial signal and the change in arterial oxygen tension (Fig. 3B). The only mechanism that would allow explaining an increase in myocardial oxygenation in dependency of a pronounced desaturation is, in fact, an increase in myocardial blood flow due to a decrease in Pao_2_.

### Cardiovascular parameters

A consistent drop of mean arterial blood pressure was monitored during the breath-holds. This may in fact be a confounder in this model, as a decreased afterload also decreases myocardial workload and thus oxygen consumption. Except for the 80/50 experiment, however, we did not observe a significant change in HR. Especially, there was no relationship between changes in myocardial SI and blood pressure or HR. Consequently, our findings suggest that in our model, myocardial oxygenation changes are dependent on changes in arterial blood gas levels.

Chilian and Layne (1990) demonstrated that arteries and arterioles exhibit significant vasodilation once the perfusion pressure decreased to 60 mmHg, with the arterioles dilating to a greater extent than the arteries. Indeed, we observed a drop in blood pressure during some of the breath-holds in our experiments. Interestingly, we observed a correlation between changes in blood pool oxygenation and HR. We cannot explain this effect as SI of the blood pool in the ventricles does not rely on perfusion or metabolic effects and is principally a representation of the Hb saturation from arterialized blood from the lungs. However, Piechnik et al. (2010) showed that an increase in HR is associated with a decrease in blood T1 that cannot be explained physiologically, indicating there may be an undetermined technical reason for this particular observation.

### Limitations

In this study, we did not measure venous blood gases from the coronary sinus. Other studies have shown that during hypercapnia saturation of the blood in the coronary sinus increased demonstrating a decrease in myocardial oxygen extraction (Wexels et al. 1985; Powers et al. 1986). Flow measurements are challenging to obtain in an MRI environment, therefore we lack the data to augment this study.

## Conclusion

Using OS CMR, we found evidence that myocardial oxygenation is preserved during hypoxia when combined with co_2_-increasing maneuvers, indicating synergistic effects of hypoxia and hypercapnia on myocardial blood flow.
